# Metabarcoding assessment of prokaryotic and eukaryotic taxa in sediments from Stellwagen Bank National Marine Sanctuary

**DOI:** 10.1038/s41598-019-51341-3

**Published:** 2019-10-15

**Authors:** Jennifer M. Polinski, John P. Bucci, Mark Gasser, Andrea G. Bodnar

**Affiliations:** 1 Gloucester Marine Genomics Institute, Inc. Gloucester, Massachusetts, USA; 2 Present Address: School of Marine Science and Ocean Engineering, University of New Hampshire, Durham, New Hampshire USA; 3 Present Address: The Johns Hopkins Applied Physics Laboratory, Laurel, Maryland USA

**Keywords:** Microbial ecology, Soil microbiology, Metagenomics, Microbial ecology

## Abstract

Stellwagen Bank National Marine Sanctuary (SBNMS) in the Gulf of Maine is a historic fishing ground renowned for remarkable productivity. Biodiversity conservation is a key management priority for SBNMS and yet data on the diversity of microorganisms, both prokaryotic and eukaryotic, is lacking. This study utilized next generation sequencing to characterize sedimentary communities within SBNMS at three sites over two seasons. Targeting 16S and 18S small subunit (SSU) rRNA genes and fungal Internal Transcribed Spacer (ITS) rDNA sequences, samples contained high diversity at all taxonomic levels and identified 127 phyla, including 115 not previously represented in the SBNMS Management Plan and Environmental Assessment. A majority of the diversity was bacterial, with 59 phyla, but also represented were nine Archaea, 18 Animalia, 14 Chromista, eight Protozoa, two Plantae, and 17 Fungi phyla. Samples from different sites and seasons were dominated by the same high abundance organisms but displayed considerable variation in rare taxa. The levels of biodiversity seen on this small spatial scale suggest that benthic communities of this area support a diverse array of micro- and macro-organisms, and provide a baseline for future studies to assess changes in community structure in response to rapid warming in the Gulf of Maine.

## Introduction

Microorganisms account for a majority of the ocean’s biomass. These marine microbes, both prokaryotic and eukaryotic, contribute vastly to ocean primary productivity and play important roles in ecosystem functions such as organic matter decomposition^1^, and cycling of carbon^2^ and nitrogen^3^. These processes can vary seasonally^1^ and influence global biogeochemical cycles^4–7^.

Advances in molecular technologies have revealed high microbial abundance and diversity in marine environments. It has been estimated that there are ~10^29^ prokaryotic cells in the open ocean^8^ and between 2–6 × 10^29^ cells in continental subseafloor sediments^9,10^. Ocean shelves (<150 m) are believed to harbor approximately 33% of the total cells in subseafloor sediments despite making up only 7% of the oceanic area^9^. Next generation sequencing (NGS) has uncovered an enormous level of phylogenetic complexity and novelty^11^. Previous studies using 16S rRNA gene diversity as a proxy for species diversity showed that shallow, coastal seafloor samples contain higher levels of bacterial diversity than deep seafloor samples^12–15^. The relatively high density and diversity of microorganisms, coupled with the accessibility of such depths, recommend ocean shelves as a primary target for surveys of sedimentary communities.

While bacterial communities have been intensely studied in recent years, less is known about diversity, distribution, and functional roles of microeukaryotes associated with marine environments. However, where applied, NGS studies have revealed previously unknown diversity of microeukaryotes, even in well-studied systems^16^. Deep sea sediments have been shown to harbor large numbers of microeukaryotes (50,000–5 million per square meter) from over 15 phyla^17,18^. 18S rRNA gene metabarcoding assessments of marine sediments also revealed distinct phylogenetic separation of shallow and deep communities, and indicated cosmopolitan deep-sea distributions for select taxa while others appear regionally restricted^19^. These studies suggest that marine microeukaryote communities are complex and diverse; however, the extent of spatial and temporal variation in community structure across marine ecosystems is still largely unknown.

The Stellwagen Bank National Marine Sanctuary (SBNMS) provides a unique environment for which baseline data of the diversity of microorganisms is lacking. Documenting patterns of microorganismal community structure and function is more important than ever under the current threat of global climate change^20,21^. SBNMS resides in the Gulf of Maine, a temperate gulf in the Northwest Atlantic Ocean that has recently experienced sea surface temperature increases faster than 99% of the global ocean^22^. Changes in ocean circulation at regional and local scales are also affecting benthic temperatures in the Gulf of Maine, with increases of 0.2 °C per decade seen from 1982 to 2014^23^. SBNMS has a long history of human use and continues to support commercially important fisheries, many of which have already felt impacts of warming^22,24^.

The most recent SBNMS Management Plan and Environmental Assessment estimated a species richness of over 575 species, but only included three genera of microorganisms^25^. This plan cites challenges with accurately evaluating the scale and consequences of changes in the sanctuary’s resource state due to a lack of baseline data for comparisons. Although several studies have since focused on pelagic microbes in the Gulf of Maine^26,27^, no studies have utilized 16S rRNA, 18S rRNA, or ITS rDNA gene metabarcoding to assess sediment microbial diversity within the boundaries of SBNMS. This study presents the first NGS metabarcoding assessment of diversity of microorganisms within SBNMS and provides much needed baseline data for future assessments and comparisons.

## Results and Discussion

### Prokaryotic diversity

**Figure 1:**
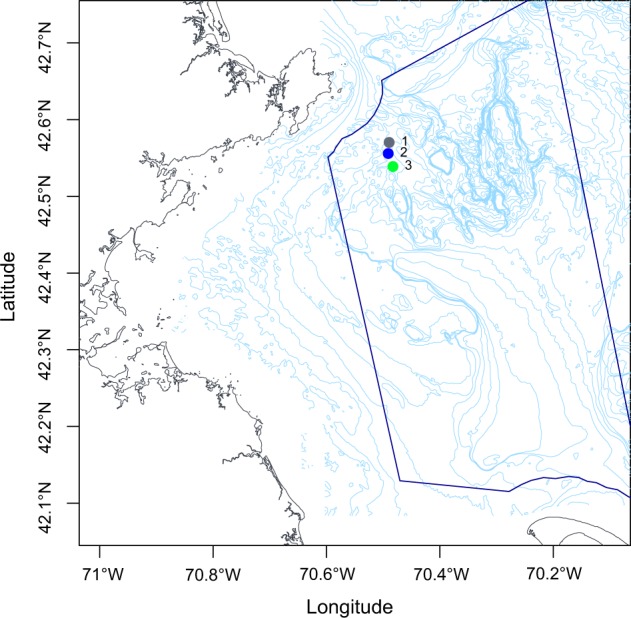
Sampling sites in Stellwagen Bank National Marine Sanctuary (SBNMS): The sanctuary boundary along the coast of Massachusetts, USA in the Atlantic Ocean is outlined in blue, and points indicate sample collection sites 1 (42°34′13.0″N 70°29′22.0″W), 2 (42°33′21.0″N 70°29′28.0″W), and 3 (42°32′20.0″N 70°28′58.0″W).

Total DNA was extracted from sediment collected from three sites (Fig. 1) within the northwest corner of SBNMS over two seasons – summer and fall. The v4 region of the 16S small subunit (SSU) rRNA gene was amplified in order to assess diversity of prokaryotic organisms present in the sediment. A total of 25,796 operational taxonomic units (OTUs) clustered at 97% sequence similarity were identified from 1,251,081 quality filtered and merged 16S rRNA gene sequencing reads. 20,732 OTUs, representing 80.48% of the sequencing reads, were classified as Bacteria, and the remaining 4,535 OTUs were classified as Archaea.

Archaea OTUs were further classified within nine phyla (Table S1). Thaumarchaeota (previously Crenarchaeota Group I) was the most abundant, represented by 21 OTUs and making up 58.65% of the archaeal reads (Table S1). Archaea in the phylum Thaumarchaeota are believed to be among the most abundant on Earth and include all ammonia-oxidizing archaea, contributing significantly to global carbon and nitrogen cycles^28^. They have been previously identified in pelagic and benthic samples in the Eastern North Atlantic^29,30^ and Western South Atlantic^31^. Bottom temperature was the driving factor for Thaumarchaeota distribution in benthic samples collected from the Artic, Northeast Atlantic, and Mediterranean basin, suggesting that they are particularly sensitive to thermal energy and will likely be impacted by global climate change^30^. Continued monitoring of benthic Thaumarchaeota community distributions within SBNMS and the wider Gulf of Maine region could provide insights on the impacts of warming on benthic prokaryotic community structure.

**Figure 2:**
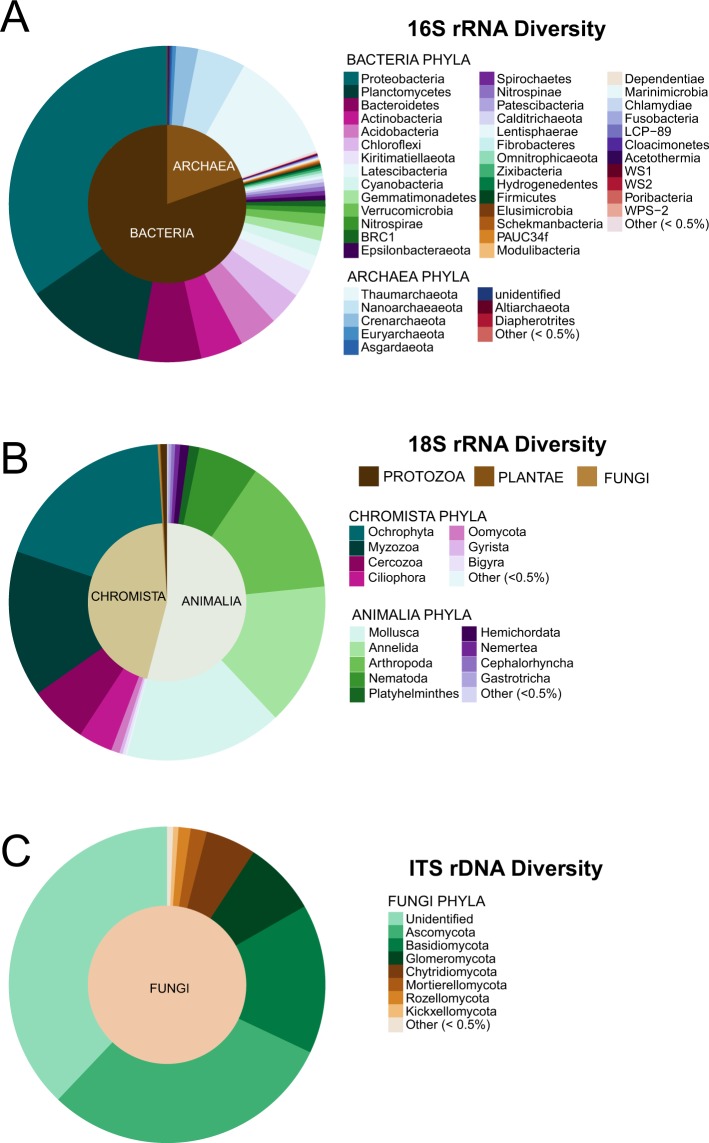
Phyla composition of samples from Stellwagen Bank National Marine Sanctuary (SBNMS): (**A**) prokaryotes identified via 16S rRNA gene sequencing, (**B**) eukaryotes identified via 18S rRNA gene sequencing, and (**C**) fungi identified via ITS rDNA sequencing in benthic sediment collected from Gloucester Basin in SBNMS.

Bacterial sequences were furthered classified within 59 phyla (Fig. 2A). The phylum Proteobacteria was the most abundant, accounting for 43% of the bacterial reads. Similar to previous studies conducted on coastal seafloor samples, the communities reported here were dominated by organisms in the classes Delta- and Gammaproteobacteria^12,15,32^. These classes made up 49.4% and 47.6%, respectively, of the Proteobacteria OTUs, with the remaining classified as Alphaproteobacteria (2.8%) and Zetaproteobacteria (0.2%). Deltaproteobacteria have been strongly associated with surficial marine sediments because many are sulfur-reducing and sulfate concentrations are highest in surface sediment layers^15,33–35^. Some taxa within Gammaproteobacteria have been suggested as drivers of sulfur oxidation and carbon fixation in coastal sediments^32,36,37^, including bacteria in the family Ectothiorhodospiraceae which accounted for ~2.6% of the Gammaproteobacteria sequences in this study.

Several classes of bacteria previously identified in benthic samples were also found in this study^12^. These include Planctomycetacia, Acidobacteria, Bacilli, and Clostridia. However, while Zinger *et al*. (2011) reported lower proportions of Planctomycetacia when comparing coastal (<200 miles from shore and <200 m depth) and deep-sea samples (>200 m), Planctomycetacia was among the more abundant classes in this study. Conversely, Zinger *et al*. reported higher proportions of Bacilli and Clostridia in coastal samples versus deep-sea, which each made up <1% of the bacterial reads here (100 m).

### Eukaryotic diversity

Relatively few studies have focused on marine microeukaryotes in comparison to bacteria. However, studies applying targeted NGS have improved understanding of diversity patterns of unicellular and microscopic multicellular eukaryotes that have been traditionally difficult to identify^38^. In this study, eukaryotic diversity in sediments from SBNMS was assessed by targeting the V2 region of the 18S SSU rRNA gene. 2,051,938 quality filtered and merged 18S rRNA sequencing reads were clustered into 8,271 OTUs at 97% sequence similarity. Eukaryotic sequences were dominated by OTUs classified in the kingdoms Animalia (54.1%) and Chromista (44.9%). These OTUs sequences were further classified into 18 Animalia and 14 Chromista phyla (Fig. 2B). Protozoa (0.6%) in eight phyla, Fungi (0.2%) in 10 phyla, and Plantae (<0.1%) in two phyla made up the remaining 18S rRNA gene reads (Table S2).

Stramenopiles, now classified in the kingdom Chromista, have been identified in marine sediments previously^19,39–41^ and comprised a majority of the non-metazoan 18S rRNA gene diversity seen in SBNMS sediment samples. Dinoflagellates (class Dinophyceae) and diatoms (class Bacillariophyceae) accounted for a majority of Chromista sequences (Table S2). Some of these organisms likely play important roles in primary production, as benthic microalgal production in SBNMS has been shown to be extensive^42^. However, the extent of microeukaryotes with potential involvement in benthic primary production is likely inflated by environmental DNA, as some 18S rRNA OTUs were classified as taxa known to be pelagic, such as the planktonic foraminifera genera *Pelagodinium* and *Protoperidinium*^43^.

Similar to previous studies of coastal benthic samples^19,40,41,44,45^, a large proportion of the 18S rRNA gene reads (54%) were assigned to metazoan taxa. The 18S rRNA primers did not exclusively target microeukaryotes, and metazoan taxa included many macro-eukaryotes such as cnidarians, sponges, and mollusks, providing additional information on benthic community composition. Environmental DNA (eDNA) originating from extracellular DNA, shed cells, or decomposing tissue of pelagic organisms likely also played a role in the abundance of metazoan taxa as several genera of macrofauna, including the commercially important fish genus *Gadus*, were represented in the OTUs.

Similar to terrestrial environments, fungi likely play important roles in detritus decomposition, particularly in suboxic and anoxic marine environments but have been considered rare in marine environments until NGS began to reveal otherwise^46^. Although reads classified as fungi only made up a small proportion of the 18S rRNA sequences, targeted sequencing with fungi-specific ITS rDNA primers revealed additional fungal diversity. A total of 2,801 OTUs were clustered at 97% similarity from 3,816,222 filtered and merged sequence reads. However, only 1,044 of these OTUs, constituting only 18% of the sequence reads, were classified as fungi. This subset of OTUs included sequences identified within 13 known fungal phyla (Fig. 2C, Table S3). Most sequences classified to phyla were Ascomycota (30.1%) and Basidiomycota (15.3%). Sequences identified within the phylum Ascomycota have also been among the most common in previous studies focusing on marine samples^47–49^.

Six of the 13 phyla identified in the ITS rDNA dataset were also found in the 18S rRNA sequences, including the five most abundant. However, four phyla identified within the 18S rRNA dataset – Zygomycota, Cryptomycota, Blastocladiomycota, and Microspoidia – were not identified within the ITS rDNA dataset. Approximately 37.9% of the sequences, represented by 425 ITS rDNA OTUs, were classified within the kingdom Fungi but were not classified to phyla due to lack of taxonomic information in the UNITE database. Similarly, a large number of OTUs in the 18S rRNA dataset were not classified to phyla due to lack of taxonomic information for the database entries that constituted the best match. Identification of divergent or previously unknown sequences occurs in many studies of marine fungi, suggesting that our understanding of fungal diversity is still lacking^47,48,50^.

### Site and season comparisons

Studying community distribution across different spatial and temporal scales is key to understanding interactions such as competition, patterns of diversity, and ecological roles^51^. Many distinct communities of both prokaryotic and eukaryotic microorganisms share a subset of similar taxa that persist over different sites or depths^15,19^. In order to assess whether OTU abundance differed over the two sampled seasons, a Wilcoxon rank test was performed using the R package ALDEx2^52^. This test was used because it can provide accurate estimates of significance for small sample sizes^53^. No significant differences were seen between the summer and fall sample sets (p-values: 0.10–1.0; Benjamini-Hochberg corrected p-values: 0.90–1.0). To assess differences between the sites, SIMPER analysis was performed with the package vegan^54^ to identify OTUs that contributed most to beta-diversity and then Kruskal-Wallis rank tests were performed to determine if OTU abundance differences were statistically significant. SIMPER identified 22, 16, and 49 contributing OTUs for the 16S rRNA, 18S rRNA, and ITS rDNA datasets, respectively, but no differences were significant between sites (p-values: 0.10–0.87).

**Figure 3:**
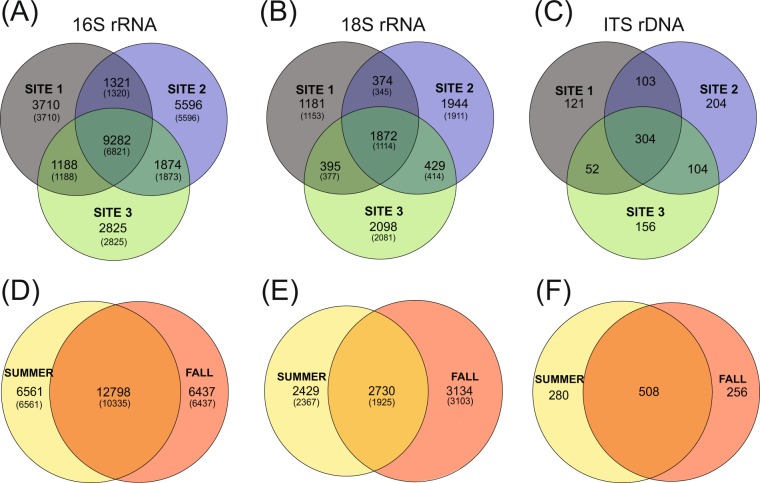
Venn diagrams showing the number of shared OTUs (**A**–**C**) between the sampling site and (**D**–**F**) the sampling seasons for (**A**,**D**) 16S rRNA gene, (**B**,**E**) 18S rRNA gene, and (**C**,**F**) ITS rDNA gene datasets. Numbers in parentheses seen in A, B, D, and E represent rare OTUs, defined here as OTUs represented by 50 or less sequence reads.

**Figure 4:**
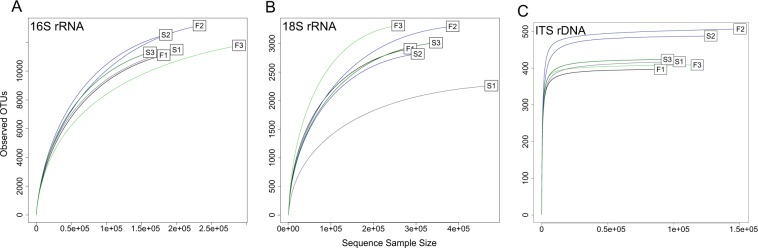
Rarefaction curves of observed OTU counts for (**A**) 16S rRNA, (**B**) 18S rRNA, and (**C**) ITS rDNA gene sequence reads. The letters S and F represent samples collected in Summer and Fall, respectively, at sites 1, 2 or 3 within Stellwagen Bank National Marine Sanctuary.

Communities within sediments of SBNMS showed low evenness, with the most abundant microorganisms found in all sites over both sampling dates but only accounting for a fraction of the diversity. Although differences in abundance were not significant, these data revealed high levels of heterogeneity among rare OTUs (<50 reads) across two seasons and three sampled locations, despite their close proximity. This heterogeneity between sites and seasons within the 16S rRNA datasets was caused by only rare OTUs, and heterogeneity in the 18S rRNA dataset was largely explained by rare OTUs (Fig. 3). Some of the variability of rare OTUs is likely due to chance based on sequencing coverage. Rarefaction curves suggest adequate coverage for all datasets (Fig. 4). However, species richness was only saturated in the ITS rDNA dataset, which consequently contained no rare OTUs, as defined here. eDNA from organisms not actively contributing to benthic community function have been shown to affect estimates of diversity in sedimentary communities^55^, although other studies testing effects of DNA from deceased organisms have found minimal effect on taxonomic diversity^56,57^. Differences in rare microorganisms could also be the result of differences in sediment physiochemical conditions, which can affect species distribution at microscopic scales^58^. Studies utilizing whole genome shotgun metagenomics and gene expression could help elucidate whether these rare microorganisms are functionally distinct.

### Summary

This study represents one of the first to utilize high-throughput NGS metabarcoding to characterize the sedimentary communities within SBNMS. The high levels of biodiversity and variability on a small spatial scale suggest that the benthic communities of this area of SBNMS are able to support a diverse array of prokaryotic and eukaryotic micro- and macro-organisms. Of the 127 phyla reported here, only 12 were included in the Preliminary Species List found in the 2010 SBNMS Management Plan and Environmental Assessment (Supplementary materials). A majority of the organisms in the management plan are metazoans, including 10 phyla identified here, with only two phyla of microorganisms represented by two genera of diatoms and one dinoflagellate in the kingdom Chromista. This is likely due to limitations of traditional morphological identification. The addition of 59 Bacteria, nine Archaea, eight Animalia, 12 Chromista, eight Protozoan, two Plantae, and 17 Fungi phyla via NGS greatly expands our knowledge of benthic biodiversity in this region and provides a baseline for future studies. However, these data represent only a small section of the protected area. More comprehensive spatial and temporal sampling will build upon this baseline to identify variation in community structure throughout SBNMS, allow comparison between sections open and closed to fishing activities, and enable identification of change caused by rapid warming in the Gulf of Maine for informed management decisions.

## Materials and Methods

Surficial sediment samples were collected at a water depth of approximately 100 m from three sites over two seasons near the northwest boundary of the Stellwagen Bank NMS under permit SBNMS-2016–005. The sampling events took place in July and October of 2016. Sampling sites were located 12–15 km off the coast of Gloucester, Massachusetts, USA and are hereafter referred to as sites 1 (42°34′13.0″N 70°29′22.0″W), 2 (42°33′21.0″N 70°29′28.0″W), and 3 (42°32′20.0″N 70°28'58.0″W; Fig. 1). Size fraction of surficial sediments at these sites was classified in the fine range category based on previous surveys^59^. A 2 L Van Veen sediment grab was used to collect the top 15 cm of benthic sediment. Fine depositional sediment was separated from pore water with a water sieve, homogenized, and transferred to a 50 mL tube. Samples were kept on ice until frozen at −20 °C the same day of collection.

Total DNA was extracted from 5 g of the fine depositional sediment using the DNeasy® PowerMax® Soil kit (Qiagen, Valencia, CA, USA). Extracted DNA was used in reactions targeting the V4 region of the bacterial 16S SSU rRNA gene, the V2 region of eukaryotic 18S SSU rRNA, and the ITS 2 region of fungal rDNA (Table 1). All primers included a linking sequence for addition of Illumina sequencing adapters and indices at their 5′ end (forward: 5′–TCGTCGGCAGCGTCAGATGTGTATAAGCAG–3′; reverse: 5′–GTCTCGTGGGCTCGGAGATGTGTATAAGAGACAG–3′). Triplicate 25 µL reactions were performed to amplify the target region using 1X Platinum™ Hot Start PCR Master Mix (ThermoFisher Scientific), 10 ng of template DNA, 0.2 µM forward primer, and 0.2 µM reverse primer. The reaction conditions were as follows: initial denaturation at 95 °C for 3 minutes, followed by 30 cycles of 95 °C for 30 sec, 55 °C for 30 sec, and 72 °C for 45 seconds, and a final extension at 72 °C for 5 minutes. Triplicate reactions were combined and PCR products were cleaned with a Monarch® PCR & DNA cleanup kit (New England Biolabs, Beverly, MA, USA). Illumina indices and sequencing adapters were ligated to cleaned PCR products using the Nextera® XT Index Kit (Illumina, San Diego, CA, USA) according to manufacturer’s instructions. Indexed amplicons were quantified with an Invitrogen™ Qubit™ fluorometer, pooled in equal concentrations, and sequenced on an Illumina MiSeq to generate 2 × 300 bp overlapping paired-end reads.

**Table 1:** Primers for initial amplification of 16S rRNA, 18S rRNA, and ITS rDNA regions.

Target	Primer Sequence (forward and reverse, respectively)	References
Bacteria & Archaea (16S)	V4_515F: 5′–GTGYCAGCMGCCGCGGTAA–3′ V4_806R-B: 5′–GGACTACNVGGGTWTCTAAT–3′	Parada *et al*.^65^ Apprill *et al*.^66^
Fungi (ITS)	ITS_86F: 5′–GTGAATCATCGAATCTTTGAA–3′ ITS_4R: 5′–TCCTCCGCTTATTGATATGC–3′	Op De Beeck *et al*.^67^
Eukaryotes (18S)	SSU_FO4: 5′–GCTTGTCTCAAAGATTAAGCC–3′ SSU_R22: 5′–GCCTGCTGCTGCCTTCCTTGGA–3′	Blaxter *et al*.^68^

For all datasets, sequencing adapters and primers were removed from the sequences. Quality-based trimming was performed with Trimmomatic version 0.38 using a 4-base wide sliding window approach with an average quality threshold of 15. Read pairs with sequences too short to allow overlap for merging were discarded (150 bp for 16S, 220 for 18S, and 180 for ITS reads, respectively). All remaining steps were performed using USEARCH version 11.0.667. Read pairs were merged and merged pairs for all samples in each dataset were pooled and dereplicated. Remaining unique sequences were clustered into Operational Taxonomic Units (OTUs) at 97% similarity and chimeric sequencers were removed using the UPARSE algorithm. Merged read pairs for each sample were then mapped to the representative OTUs to determine OTU abundance per sample. OTUs represented by a single sequence were discarded. Blastn was used to determine OTU taxonomy, with an e-value cutoff of 1e10^−4^ and only the best hit reported. 16S rRNA OTUs were classified using a combination of the SILVA v132^60^ and RDP databases^61^. 18S rRNA OTUs were classified with SILVA v132 and PR2 v4.10.0^62^ databases, with taxonomy of organisms in the kingdom Chromista following that accepted by the World Register of Marine Species^63^. ITS rDNA OTUs were classified with the UNITE database^64^, and any sequences not classified in the kingdom Fungi were discarded.

## Supplementary information

Supplmentary Material

## Data Availability

All raw sequencing data from this study have been deposited in the NCBI Sequence Read Archive (SRA) under BioProject accession number PRJNA517501 (BioSample accessions SAMN10834666–SAMN10834683).
