# Supplementary material for: Metabarcoding assessment of prokaryotic and eukaryotic taxa in sediments from Stellwagen Bank National Marine Sanctuary
Source: Sci Rep. 2019 Oct 15;9:14820. doi: 10.1038/s41598-019-51341-3 (PMC6794287; doi:10.1038/s41598-019-51341-3)
Supplement: Supplementary file 1 — Supplmentary Material [file 41598_2019_51341_MOESM1_ESM.pdf]

Metabarcoding assessment of prokaryotic and eukaryotic taxa in sediments from Stellwagen Bank National Marine Sanctuary

Supplementary Materials

Jennifer M. Polinski[1,*], John P. Bucci[1,2], Mark Gasser[1,3], Andrea G. Bodnar[1]

[1] Gloucester Marine Genomics Institute, Inc. Gloucester, Massachusetts, USA

[2] Current address: School of Marine Science and Ocean Engineering, University of New Hampshire, Durham, New Hampshire, USA

[3] The Johns Hopkins Applied Physics Laboratory, Laurel, Maryland, USA

* Corresponding author

Email: jennifer.polinski@gmgi.org

Keywords: metabarcoding, diversity, benthic, 16S rRNA, 18S rRNA, ITS rDNA

Supplementary Table S1: Total number of OTUs and sequence read abundance for each class identified within the prokaryotic 16S-v4 SSU rRNA dataset.

Target region	Kingdom	Phylum	Class	Number of OTUs	Total Abundance
16S-v4	Bacteria	Acetothermia	Acetothermiia	16	736
16S-v4	Bacteria	Acidobacteria	Thermoanaerobaculia	307	25035
16S-v4	Bacteria	Acidobacteria	Subgroup 22	280	10097
16S-v4	Bacteria	Acidobacteria	Aminicenantia	83	2938
16S-v4	Bacteria	Acidobacteria	Subgroup 17	47	2543
16S-v4	Bacteria	Acidobacteria	Subgroup 21	38	2483
16S-v4	Bacteria	Acidobacteria	Subgroup 26	19	1152
16S-v4	Bacteria	Acidobacteria	Acidobacteriia	44	949
16S-v4	Bacteria	Acidobacteria	Subgroup 6	38	849
16S-v4	Bacteria	Acidobacteria	Subgroup 9	32	809
16S-v4	Bacteria	Acidobacteria	AT-s3-28	62	727
16S-v4	Bacteria	Acidobacteria	Subgroup 18	18	467
16S-v4	Bacteria	Acidobacteria	Holophagae	34	436
16S-v4	Bacteria	Acidobacteria	Blastocatellia (Subgroup 4)	7	290
16S-v4	Bacteria	Acidobacteria	d142	18	233
16S-v4	Bacteria	Acidobacteria	Subgroup 5	15	148
16S-v4	Bacteria	Acidobacteria	Subgroup 11	5	76
16S-v4	Bacteria	Acidobacteria	Subgroup 19	2	27
16S-v4	Bacteria	Acidobacteria	c5LKS83	6	25
16S-v4	Bacteria	Acidobacteria	ODP1230B23.02	3	21
16S-v4	Bacteria	Actinobacteria	Acidimicrobiia	372	51344
16S-v4	Bacteria	Actinobacteria	WCHB1-81	6	1233
16S-v4	Bacteria	Actinobacteria	Thermoleophilia	27	1100
16S-v4	Bacteria	Actinobacteria	Actinobacteria	23	308
16S-v4	Bacteria	Actinobacteria	Coriobacteriia	14	141
16S-v4	Bacteria	Actinobacteria	MB-A2-108	5	51
16S-v4	Bacteria	Actinobacteria	RBG-16-55-12	2	16
16S-v4	Bacteria	Actinobacteria	Nitriliruptoria	2	4
16S-v4	Bacteria	Aegiribacteria	unclassified	7	111
16S-v4	Bacteria	Aerophobetes	unclassified	2	13
16S-v4	Bacteria	AncK6	unclassified	9	353
16S-v4	Bacteria	Armatimonadetes	DG-56	9	85
16S-v4	Bacteria	Armatimonadetes	Fimbriimonadia	3	52
16S-v4	Bacteria	Armatimonadetes	unclassified	5	28
16S-v4	Bacteria	Atribacteria	JS1	3	94
16S-v4	Bacteria	Bacteroidetes	Bacteroidia	949	72416
16S-v4	Bacteria	Bacteroidetes	Ignavibacteria	76	6184
16S-v4	Bacteria	Bacteroidetes	Rhodothermia	41	1623
16S-v4	Bacteria	BRC1	unclassified	327	7607
16S-v4	Bacteria	BRC1	candidate division BRC1	10	185
16S-v4	Bacteria	BRC1	Omnitrophica	1	12
16S-v4	Bacteria	Calditrichaeota	Calditrichia	125	4586

16S-v4	Bacteria	Chlamydiae	LD1-PA32	58	716
16S-v4	Bacteria	Chlamydiae	Chlamydiae	101	289
16S-v4	Bacteria	Chloroflexi	Anaerolineae	612	36138
16S-v4	Bacteria	Chloroflexi	Dehalococcoidia	259	4961
16S-v4	Bacteria	Chloroflexi	KD4-96	24	1134
16S-v4	Bacteria	Chloroflexi	JG30-KF-CM66	7	184
16S-v4	Bacteria	Chloroflexi	Chloroflexia	2	82
16S-v4	Bacteria	Chloroflexi	TK17	2	65
16S-v4	Bacteria	Chloroflexi	N9D0	3	37
16S-v4	Bacteria	Chloroflexi	Ktedonobacteria	4	10
16S-v4	Bacteria	Chloroflexi	OLB14	1	4
16S-v4	Bacteria	Chloroflexi	TK10	1	2
16S-v4	Bacteria	Chloroflexi	Gitt-GS-136	1	2
16S-v4	Bacteria	Chloroflexi	AD3	1	2
16S-v4	Bacteria	CK-2C2-2	unclassified	27	315
16S-v4	Bacteria	CK-2C2-2	Candidatus Cloacimonetes	3	14
16S-v4	Bacteria	Cloacimonetes	Cloacimonadia	15	765
16S-v4	Bacteria	Cyanobacteria	Oxyphotobacteria	18	19698
16S-v4	Bacteria	Cyanobacteria	Melainabacteria	68	68
16S-v4	Bacteria	Cyanobacteria	Sericytochromatia	9	36
16S-v4	Bacteria	Dadabacteria	Dadabacteriia	9	427
16S-v4	Bacteria	Deinococcus-Thermus	Deinococci	2	55
16S-v4	Bacteria	Dependentiae	Babeliae	203	1121
16S-v4	Bacteria	Elusimicrobia	Elusimicrobia	77	557
16S-v4	Bacteria	Elusimicrobia	Lineage II	57	534
16S-v4	Bacteria	Elusimicrobia	Rs-M47	51	439
16S-v4	Bacteria	Elusimicrobia	Endomicrobia	27	136
16S-v4	Bacteria	Elusimicrobia	43584	7	39
16S-v4	Bacteria	Elusimicrobia	unclassified	1	3
16S-v4	Bacteria	Entotheonellaeota	Entotheonellia	2	14
16S-v4	Bacteria	Epsilonbacteraeota	Campylobacteria	45	6307
16S-v4	Bacteria	FBP	Armatimonadetes	2	11
16S-v4	Bacteria	FCPU426	unclassified	22	198
16S-v4	Bacteria	Fibrobacteres	Fibrobacteria	155	2865
16S-v4	Bacteria	Fibrobacteres	Chitinivibrionia	29	617
16S-v4	Bacteria	Firmicutes	Clostridia	143	2053
16S-v4	Bacteria	Firmicutes	Bacilli	19	185
16S-v4	Bacteria	Firmicutes	Negativicutes	3	96
16S-v4	Bacteria	Firmicutes	Erysipelotrichia	1	10
16S-v4	Bacteria	Firmicutes	unclassified	2	5
16S-v4	Bacteria	Fusobacteria	Fusobacteriia	13	1002
16S-v4	Bacteria	Gemmatimonadetes	BD2-11	129	9168
16S-v4	Bacteria	Gemmatimonadetes	PAUC43f	152	7503
16S-v4	Bacteria	Gemmatimonadetes	Gemmatimonadetes	33	2422
16S-v4	Bacteria	Gemmatimonadetes	MD2902-B12	19	385
16S-v4	Bacteria	Gemmatimonadetes	S0134	1	26
16S-v4	Bacteria	Gemmatimonadetes	Longimicrobia	1	2

16S-v4	Bacteria	GN01	unclassified	4	106
16S-v4	Bacteria	Halanaerobiaeota	Halanaerobiia	2	15
16S-v4	Bacteria	Hydrogenedentes	Hydrogenedentia	147	2547
16S-v4	Bacteria	Kiritimatiellaeota	Kiritimatiellae	827	35401
16S-v4	Bacteria	Latescibacteria	unclassified	301	13344
16S-v4	Bacteria	Latescibacteria	Latescibacteria	177	7014
16S-v4	Bacteria	LCP-89	unclassified	73	984
16S-v4	Bacteria	LCP-89	Calditrichaeota	2	12
16S-v4	Bacteria	Lentisphaerae	Oligosphaeria	110	2824
16S-v4	Bacteria	Lentisphaerae	Lentisphaeria	92	949
16S-v4	Bacteria	Margulisbacteria	unclassified	94	295
16S-v4	Bacteria	Margulisbacteria	Candidatus Margulisbacteria	15	43
16S-v4	Bacteria	Marinimicrobia	SAR406 clade	40	1039
16S-v4	Bacteria	MAT-CR-M4-B07	unclassified	1	4
16S-v4	Bacteria	Modulibacteria	Moduliflexia	34	1290
16S-v4	Bacteria	Nitrospinae	P9X2b3D02	60	3623
16S-v4	Bacteria	Nitrospinae	Nitrospinia	37	1943
16S-v4	Bacteria	Nitrospirae	Nitrospira	28	5134
16S-v4	Bacteria	Nitrospirae	Thermodesulfovibrionia	73	3564
16S-v4	Bacteria	Nitrospirae	BMS9AB35	21	108
16S-v4	Bacteria	Nitrospirae	37010	3	9
16S-v4	Bacteria	Omnitrophicaeota	unclassified	306	2211
16S-v4	Bacteria	Omnitrophicaeota	Omnitrophia	117	1120
16S-v4	Bacteria	Omnitrophicaeota	Omnitrophica	6	51
16S-v4	Bacteria	Patescibacteria	Gracilibacteria	279	2089
16S-v4	Bacteria	Patescibacteria	Parcubacteria	99	952
16S-v4	Bacteria	Patescibacteria	ABY1	165	898
16S-v4	Bacteria	Patescibacteria	Saccharimonadia	51	827
16S-v4	Bacteria	Patescibacteria	Microgenomatia	58	312
16S-v4	Bacteria	Patescibacteria	WWE3	28	144
16S-v4	Bacteria	Patescibacteria	Berkelbacteria	4	26
16S-v4	Bacteria	Patescibacteria	CPR2	4	21
16S-v4	Bacteria	Patescibacteria	Kazania	4	15
16S-v4	Bacteria	Patescibacteria	MD2896-B216	1	3
16S-v4	Bacteria	Planctomycetes	Phycisphaerae	1429	65476
16S-v4	Bacteria	Planctomycetes	Planctomycetacia	896	46388
16S-v4	Bacteria	Planctomycetes	OM190	518	15987
16S-v4	Bacteria	Planctomycetes	Pla4 lineage	270	9103
16S-v4	Bacteria	Planctomycetes	vadinHA49	182	6740
16S-v4	Bacteria	Planctomycetes	Pla3 lineage	257	4375
16S-v4	Bacteria	Planctomycetes	Brocadiae	66	3587
16S-v4	Bacteria	Planctomycetes	BD7-11	133	2581
16S-v4	Bacteria	Planctomycetes	SPG12-343-353-B69	23	710
16S-v4	Bacteria	Planctomycetes	ODP123	17	269
16S-v4	Bacteria	Planctomycetes	SGST604	6	89
16S-v4	Bacteria	Planctomycetes	028H05-P-BN-P5	9	64
16S-v4	Bacteria	Poribacteria	unclassified	17	543

16S-v4	Bacteria	Proteobacteria	Deltaproteobacteria	3785	213425
16S-v4	Bacteria	Proteobacteria	Gammaproteobacteria	2854	205496
16S-v4	Bacteria	Proteobacteria	Alphaproteobacteria	476	12114
16S-v4	Bacteria	Proteobacteria	Zetaproteobacteria	19	855
16S-v4	Bacteria	Proteobacteria	Magnetococcia	13	50
16S-v4	Bacteria	Rokubacteria	NC10	5	30
16S-v4	Bacteria	Schekmanbacteria	unclassified	41	1628
16S-v4	Bacteria	Spirochaetes	Spirochaetia	272	4924
16S-v4	Bacteria	Spirochaetes	Leptospirae	71	623
16S-v4	Bacteria	Spirochaetes	V2072-189E03	12	125
16S-v4	Bacteria	Spirochaetes	unclassified	12	70
16S-v4	Bacteria	Spirochaetes	Brachyspirae	2	26
16S-v4	Bacteria	Spirochaetes	MVP-15	2	13
16S-v4	Bacteria	TA06	unclassified	7	124
16S-v4	Bacteria	Tenericutes	Mollicutes	13	113
16S-v4	Bacteria	Verrucomicrobia	Verrucomicrobiae	271	16754
16S-v4	Bacteria	WOR-1	unclassified	27	231
16S-v4	Bacteria	WPS-2	unclassified	30	493
16S-v4	Bacteria	WS1	unclassified	28	600
16S-v4	Bacteria	WS2	unclassified	29	555
16S-v4	Bacteria	WS4	unclassified	1	27
16S-v4	Bacteria	Zixibacteria	unclassified	147	3032
16S-v4	Archaea	Altiarchaeota	Altiarchaeia	109	1631
16S-v4	Archaea	Asgardaeota	Lokiarchaeia	20	1416
16S-v4	Archaea	Asgardaeota	Odinarchaeia	19	522
16S-v4	Archaea	Asgardaeota	Heimdallarchaeia	11	238
16S-v4	Archaea	Asgardaeota	unclassified	3	114
16S-v4	Archaea	Crenarchaeota	Bathyarchaeia	91	28042
16S-v4	Archaea	Diapherotrites	Micrarchaeia	21	351
16S-v4	Archaea	Diapherotrites	Iainarchaeia	33	141
16S-v4	Archaea	Euryarchaeota	Thermoplasmata	120	3707
16S-v4	Archaea	Euryarchaeota	Thermococci	47	780
16S-v4	Archaea	Euryarchaeota	Methanomicrobia	17	128
16S-v4	Archaea	Euryarchaeota	Methanobacteria	10	42
16S-v4	Archaea	Euryarchaeota	Methanococci	1	16
16S-v4	Archaea	Euryarchaeota	Archaeoglobi	1	2
16S-v4	Archaea	Hadesarchaeaeota	unclassified	3	43
16S-v4	Archaea	Hydrothermarchaeota	unclassified	6	58
16S-v4	Archaea	Nanoarchaeaeota	Woesearchaeia	3901	60826
16S-v4	Archaea	Nanoarchaeaeota	Nanohaloarchaeia	90	613
16S-v4	Archaea	Thaumarchaeota	Nitrososphaeria	17	142387
16S-v4	Archaea	Thaumarchaeota	unclassified	3	382
16S-v4	Archaea	Thaumarchaeota	SCGC	1	58
16S-v4	Archaea	unclassified	unclassified	11	2035

Supplementary Table S2: Total number of OTUs and sequence read abundance for each class identified within the eukaryotic 18S-v2 SSU rRNA dataset.

Target region	Kingdom	Phylum	Class	Number of OTUs	Total Abundance
18S-v2	Animalia	Annelida	Polychaeta	260	299541
18S-v2	Animalia	Annelida	unclassified	6	93
18S-v2	Animalia	Annelida	Clitellata	4	67
18S-v2	Animalia	Arthropoda	Hexanauplia	89	162643
18S-v2	Animalia	Arthropoda	Ostracoda	40	82960
18S-v2	Animalia	Arthropoda	Arachnida	47	39973
18S-v2	Animalia	Arthropoda	Crustacea-unclassified	16	159
18S-v2	Animalia	Arthropoda	Branchiopoda	10	30
18S-v2	Animalia	Arthropoda	Insecta	4	19
18S-v2	Animalia	Arthropoda	Collembola	4	9
18S-v2	Animalia	Arthropoda	Malacostraca	1	5
18S-v2	Animalia	Brachiopoda	Craniata	6	484
18S-v2	Animalia	Brachiopoda	Lingulata	11	272
18S-v2	Animalia	Brachiopoda	Rhynchonellata	4	21
18S-v2	Animalia	Brachiopoda	Phoronida	2	7
18S-v2	Animalia	Bryozoa	Gymnolaemata	1	71
18S-v2	Animalia	Bryozoa	Phylactolaemata	2	25
18S-v2	Animalia	Cephalorhyncha	Kinorhyncha	12	7666
18S-v2	Animalia	Cephalorhyncha	Priapulida	4	14
18S-v2	Animalia	Chordata	Ascidiacea	6	157
18S-v2	Animalia	Chordata	Thaliacea	1	22
18S-v2	Animalia	Chordata	Appendicularia	2	15
18S-v2	Animalia	Chordata	Actinopterygii	2	11
18S-v2	Animalia	Cnidaria	Hydrozoa	17	371
18S-v2	Animalia	Cnidaria	Anthozoa	2	5
18S-v2	Animalia	Cnidaria	Scyphozoa	2	5
18S-v2	Animalia	Entoprocta	Solitaria	2	204
18S-v2	Animalia	Gastrotricha	Chaetonotida	33	6118
18S-v2	Animalia	Hemichordata	Enteropneusta	26	18377
18S-v2	Animalia	Mollusca	Bivalvia	51	329586
18S-v2	Animalia	Mollusca	Gastropoda	5	381
18S-v2	Animalia	Mollusca	Polyplacophora	1	2
18S-v2	Animalia	Nematoda	Chromadorea	751	98697
18S-v2	Animalia	Nematoda	Enoplea	140	28133
18S-v2	Animalia	Nematoda	unclassified	5	100
18S-v2	Animalia	Nemertea	Hoplonemertea	19	8198
18S-v2	Animalia	Nemertea	Palaeonemertea	12	2214
18S-v2	Animalia	Nemertea	Pilidiophora	1	34
18S-v2	Animalia	Placozoa	Placozoa (family Trichoplacidae)	2	5
18S-v2	Animalia	Platyhelminthes	Rhabditophora	56	21488
18S-v2	Animalia	Platyhelminthes	Cestoda	2	62
18S-v2	Animalia	Platyhelminthes	Trematoda	1	2

18S-v2	Animalia	Porifera	Demospongiae	4	21
18S-v2	Animalia	Porifera	Calcarea	3	9
18S-v2	Animalia	Rotifera	Eurotatoria	4	105
18S-v2	Animalia	Rotifera	unclassified	2	8
18S-v2	Animalia	Xenacoelomorpha	Acoelomorpha	20	1081
18S-v2	Chromista	Bigyra	Labyrinthulea	209	4695
18S-v2	Chromista	Bigyra	MAST-9	15	1092
18S-v2	Chromista	Bigyra	MAST-8	34	198
18S-v2	Chromista	Bigyra	MAST-6	22	138
18S-v2	Chromista	Bigyra	MAST-4	7	126
18S-v2	Chromista	Bigyra	Bicoecea	11	46
18S-v2	Chromista	Bigyra	MAST-7	3	8
18S-v2	Chromista	Centroheliozoa	Centroheliozoa (order Pterocystida)	14	51
18S-v2	Chromista	Centroheliozoa	unclassified	6	15
18S-v2	Chromista	Cercozoa	Thecofilosea	1334	83879
18S-v2	Chromista	Cercozoa	Imbricatea	452	35097
18S-v2	Chromista	Cercozoa	Phytomyxea	34	816
18S-v2	Chromista	Cercozoa	unclassified	82	670
18S-v2	Chromista	Cercozoa	Ascetosporea	21	604
18S-v2	Chromista	Cercozoa	Sarcomonadea	73	597
18S-v2	Chromista	Cercozoa	Proteomyxidea	65	566
18S-v2	Chromista	Cercozoa	Granofilosea	65	352
18S-v2	Chromista	Cercozoa	Novel-clade-10-12	35	200
18S-v2	Chromista	Cercozoa	Novel Clade 12	6	32
18S-v2	Chromista	Cercozoa	Conoidasida	13	32
18S-v2	Chromista	Cercozoa	Chlorarachniophyceae	9	29
18S-v2	Chromista	Cercozoa	Filosa-class	3	17
18S-v2	Chromista	Cercozoa	Endomyxa Novel Clade 9	3	13
18S-v2	Chromista	Cercozoa	Phaeodarea	1	7
18S-v2	Chromista	Cercozoa	Metromonadea	1	2
18S-v2	Chromista	Choanozoa	Choanoflagellida	2	4
18S-v2	Chromista	Ciliophora	Oligotrichea	209	69505
18S-v2	Chromista	Ciliophora	Litostomatea	31	1211
18S-v2	Chromista	Ciliophora	Spirotrichea	57	532
18S-v2	Chromista	Ciliophora	Oligohymenophorea	23	128
18S-v2	Chromista	Ciliophora	Karyorelictea	10	60
18S-v2	Chromista	Ciliophora	Cariacotrichea	10	40
18S-v2	Chromista	Ciliophora	Prostomatea	5	33
18S-v2	Chromista	Ciliophora	Heterotrichea	4	18
18S-v2	Chromista	Ciliophora	CONTH_6	3	9
18S-v2	Chromista	Ciliophora	Colpodea	1	2
18S-v2	Chromista	Cryptista	Palpitia	1	3
18S-v2	Chromista	Cryptophyta	Telonemea	10	145
18S-v2	Chromista	Cryptophyta	Katablepharidaceae	19	109
18S-v2	Chromista	Cryptophyta	Cryptophyceae	13	72
18S-v2	Chromista	Gyrista	MAST-1	15	4597

18S-v2	Chromista	Gyrista	MAST-12	49	1949
18S-v2	Chromista	Gyrista	Hyphochytriomyceta	30	199
18S-v2	Chromista	Gyrista	MAST-3	17	84
18S-v2	Chromista	Gyrista	MAST-2	2	4
18S-v2	Chromista	Haptophyta	Prymnesiophyceae	31	1380
18S-v2	Chromista	Haptophyta	Pavlovophyceae	1	3
18S-v2	Chromista	Haptophyta	Haptophyta_Clade_HAP4	1	3
18S-v2	Chromista	Haptophyta	Haptophyta_Clade_HAP5	1	3
18S-v2	Chromista	Haptophyta	Haptophyta_Clade_HAP2	1	2
18S-v2	Chromista	Myzozoa	Dinophyceae	1329	283228
18S-v2	Chromista	Myzozoa	Conoidasida	189	22967
18S-v2	Chromista	Myzozoa	Apicomonadea	32	752
18S-v2	Chromista	Myzozoa	Perkinsea	21	265
18S-v2	Chromista	Myzozoa	unclassified	4	52
18S-v2	Chromista	Myzozoa	Aconoidasida	1	35
18S-v2	Chromista	Myzozoa	Apicomplexa-class	1	18
18S-v2	Chromista	Ochrophyta	Bacillariophyceae	702	358022
18S-v2	Chromista	Ochrophyta	Pelagophyceae	38	15172
18S-v2	Chromista	Ochrophyta	Mediophyceae	23	8098
18S-v2	Chromista	Ochrophyta	Chrysophyceae	77	2726
18S-v2	Chromista	Ochrophyta	Dictyochophyceae	18	927
18S-v2	Chromista	Ochrophyta	MOCH-2	15	729
18S-v2	Chromista	Ochrophyta	Bolidophyceae	42	413
18S-v2	Chromista	Ochrophyta	Xanthophyceae	1	235
18S-v2	Chromista	Ochrophyta	Raphidophyceae	13	144
18S-v2	Chromista	Ochrophyta	Synurophyceae	9	55
18S-v2	Chromista	Ochrophyta	MOCH-5	2	40
18S-v2	Chromista	Ochrophyta	Phaeothamniophyceae	3	24
18S-v2	Chromista	Ochrophyta	Phaeophyceae	2	5
18S-v2	Chromista	Oomycota	Peronosporea	102	17082
18S-v2	Chromista	Oomycota	Hyphochytrea	24	438
18S-v2	Chromista	Oomycota	unclassified	8	124
18S-v2	Chromista	Oomycota	Oomycota (order Haptoglossales)	1	6
18S-v2	Chromista	Oomycota	Bigyromonadea	1	2
18S-v2	Chromista	Protalveolata	Colponemea	4	14
18S-v2	Chromista	Radiolaria	RAD-B	21	821
18S-v2	Chromista	Radiolaria	unclassified	5	489
18S-v2	Chromista	Radiolaria	Polycystinea	5	83
18S-v2	Chromista	Radiolaria	RAD-C	1	7
18S-v2	Chromista	Radiolaria	Acantharea	1	5
18S-v2	Chromista	Rhizaria-unclassified	unclassified	1	5
18S-v2	Chromista	Stramenopiles	Labyrinthulomycetes	3	18
18S-v2	Chromista	unclassified	unclassified	16	201
18S-v2	Fungi	Ascomycota	Leotiomycetes	12	283
18S-v2	Fungi	Ascomycota	Saccharomycetes	18	163
18S-v2	Fungi	Ascomycota	Schizosaccharomycetes	7	160

18S-v2	Fungi	Ascomycota	Dothideomycetes	11	77
18S-v2	Fungi	Ascomycota	Sordariomycetes	9	30
18S-v2	Fungi	Ascomycota	Eurotiomycetes	4	16
18S-v2	Fungi	Ascomycota	Taphrinomycetes	2	4
18S-v2	Fungi	Basidiobolomycota	Basidiobolomycetes	3	13
18S-v2	Fungi	Basidiomycota	Agaricomycetes	14	39
18S-v2	Fungi	Basidiomycota	Tremellomycetes	6	34
18S-v2	Fungi	Basidiomycota	Exobasidiomycetes	4	14
18S-v2	Fungi	Basidiomycota	Dacrymycetes	2	5
18S-v2	Fungi	Basidiomycota	Microbotryomycetes	1	3
18S-v2	Fungi	Blastocladiomycota	Blastocladiomycetes	2	34
18S-v2	Fungi	Chytridiomycota	unclassified	39	1758
18S-v2	Fungi	Chytridiomycota	Chytridiomycetes	65	1146
18S-v2	Fungi	Chytridiomycota	Spizellomycetes	16	63
18S-v2	Fungi	Chytridiomycota	Lobulomycetes	1	3
18S-v2	Fungi	Chytridiomycota	Chytridiomycota (order Lagenidiales)	1	2
18S-v2	Fungi	Cryptomycota	LKM11	9	29
18S-v2	Fungi	Cryptomycota	unclassified	3	6
18S-v2	Fungi	Cryptomycota	Cryptomycota Incertae Sedis	1	4
18S-v2	Fungi	Glomeromycota	Glomeromycetes	3	7
18S-v2	Fungi	Glomeromycota	Archaeosporomycetes	3	7
18S-v2	Fungi	Glomeromycota	Paraglomeromycetes	1	3
18S-v2	Fungi	Microsporidia	Microsporea	1	9
18S-v2	Fungi	Mortierellomycota	unclassified	5	33
18S-v2	Fungi	Mortierellomycota	Mortierellomycetes	3	14
18S-v2	Fungi	Zygomycota	Neocallimastigomycetes	20	422
18S-v2	Fungi	Zygomycota	Mortierellomycotina	4	25
18S-v2	Fungi	Zygomycota	Mucoromycetes	1	3
18S-v2	Fungi	unclassified	unclassified	32	152
18S-v2	Plantae	Chlorophyta	Chlorophyta	97	1486
18S-v2	Plantae	Chlorophyta	Chlorophyceae	14	268
18S-v2	Plantae	Chlorophyta	Mamiellophyceae	2	52
18S-v2	Plantae	Chlorophyta	Prasinophyceae	1	8
18S-v2	Plantae	Chlorophyta	Prasinophytae	2	6
18S-v2	Plantae	Chlorophyta	unclassified	1	3
18S-v2	Plantae	Rhodophyta	Stylonematophyceae	1	28
18S-v2	Plantae	Rhodophyta	Bangiophyceae	1	6
18S-v2	Plantae	Rhodophyta	Florideophyceae	1	2
18S-v2	Plantae	unclassified	unclassified	15	128
18S-v2	Protozoa	Amoebozoa	Discosea	33	178
18S-v2	Protozoa	Amoebozoa	Breviatea	11	50
18S-v2	Protozoa	Amoebozoa	Tubulinea	13	42
18S-v2	Protozoa	Amoebozoa	Variosea	6	15
18S-v2	Protozoa	Amoebozoa	Mycetozoa	1	2
18S-v2	Protozoa	Amoebozoa	Mycamoeba-lineage	1	2
18S-v2	Protozoa	Apusozoa	Thecomonadea	89	1128

18S-v2	Protozoa	Apusozoa	Hilomonadea	6	15
18S-v2	Protozoa	Choanozoa	Choanoflagellatea	24	174
18S-v2	Protozoa	Choanozoa	Ichthyosporea	20	69
18S-v2	Protozoa	Choanozoa	Cristidiscoidea	9	27
18S-v2	Protozoa	Choanozoa	unclassified	3	6
18S-v2	Protozoa	Loukozoa	Jakobea	7	369
18S-v2	Protozoa	Metamonada	Carpediemonadea	5	101
18S-v2	Protozoa	Metamonada	Fornicata	1	3
18S-v2	Protozoa	Metamonada	Preaxostyla	1	2
18S-v2	Protozoa	Picozoa	unclassified	3	12
18S-v2	Protozoa	Protozoa incertae sedis (Genus Kiitoksia)	Protozoa incertae sedis (Genus Kiitoksia)	3	14
18S-v2	Protozoa	Protozoa incertae sedis (Genus Pirsonia)	Pirsoniales	120	11121
18S-v2	Protozoa	unclassified	unclassified	3	11

Supplementary Table S3: Total number of OTUs and sequence read abundance for each class identified within the fungal ITS-2 SSU rDNA dataset.

Target region	Kingdom	Phylum	Class	Number of OTUs	Total Abundance
ITS-2	Fungi	Aphelidiomycota	unclassified	1	91
ITS-2	Fungi	Ascomycota	Sordariomycetes	86	60058
ITS-2	Fungi	Ascomycota	Dothideomycetes	37	48912
ITS-2	Fungi	Ascomycota	Eurotiomycetes	53	33869
ITS-2	Fungi	Ascomycota	Leotiomycetes	23	25004
ITS-2	Fungi	Ascomycota	Saccharomycetes	33	23747
ITS-2	Fungi	Ascomycota	unclassified	16	5847
ITS-2	Fungi	Ascomycota	Pezizomycetes	6	4268
ITS-2	Fungi	Ascomycota	Lecanoromycetes	2	2365
ITS-2	Fungi	Ascomycota	Laboulbeniomycetes	1	958
ITS-2	Fungi	Ascomycota	Orbiliomycetes	2	561
ITS-2	Fungi	Ascomycota	Neolectomycetes	1	358
ITS-2	Fungi	Ascomycota	Pezizomycotina	1	195
ITS-2	Fungi	Basidiobolomycota	Basidiobolomycetes	1	611
ITS-2	Fungi	Basidiomycota	Agaricomycetes	156	79405
ITS-2	Fungi	Basidiomycota	Tremellomycetes	16	10502
ITS-2	Fungi	Basidiomycota	unclassified	12	4854
ITS-2	Fungi	Basidiomycota	Microbotryomycetes	8	3865
ITS-2	Fungi	Basidiomycota	Tritirachiomycetes	2	1710
ITS-2	Fungi	Basidiomycota	Pucciniomycetes	7	1555
ITS-2	Fungi	Basidiomycota	Exobasidiomycetes	3	1300
ITS-2	Fungi	Basidiomycota	Ustilaginomycetes	4	949
ITS-2	Fungi	Basidiomycota	Agaricostilbomycetes	1	310
ITS-2	Fungi	Basidiomycota	Malasseziomycetes	1	264
ITS-2	Fungi	Chytridiomycota	Rhizophydiomycetes	25	15366
ITS-2	Fungi	Chytridiomycota	unclassified	7	14957
ITS-2	Fungi	Chytridiomycota	Spizellomycetes	6	4986
ITS-2	Fungi	Entomophthoromycota	unclassified	1	220
ITS-2	Fungi	Glomeromycota	Glomeromycetes	29	49236
ITS-2	Fungi	Glomeromycota	unclassified	4	2440
ITS-2	Fungi	Kickxellomycota	Harpellomycetes	8	2805
ITS-2	Fungi	Kickxellomycota	Kickxellomycetes	2	917
ITS-2	Fungi	Monoblepharomycota	Monoblepharidomycetes	1	119
ITS-2	Fungi	Mortierellomycota	Mortierellomycetes	29	9257
ITS-2	Fungi	Mortierellomycota	unclassified	6	2081
ITS-2	Fungi	Mucoromycota	Umbelopsidomycetes	3	2003
ITS-2	Fungi	Mucoromycota	Mucoromycetes	3	1040
ITS-2	Fungi	Neocallimastigomycota	Neocallimastigomycetes	1	113
ITS-2	Fungi	Rozellomycota	Rozellomycotina incertae sedis	13	5400
ITS-2	Fungi	Rozellomycota	unclassified	8	3090
ITS-2	Fungi	unclassified	unclassified	425	260001
